# Effect of herbal medicine *Bojungikgi-tang* on gut microbiome and symptoms in anorexic patients with atopic dermatitis: a randomized controlled trial

**DOI:** 10.3389/fphar.2025.1593477

**Published:** 2025-06-03

**Authors:** Boram Lee, Mi Mi Ko, You Mee Ahn, Hyo-Ju Park, So Young Jung, Hyun-A Jung, Hesol Lee, Pyung-Wha Kim, Yujin Choi, Kyungsun Han, Sarah Shin, Jeeyoun Jung

**Affiliations:** ^1^ KM Science Research Division, Korea Institute of Oriental Medicine, Daejeon, Republic of Korea; ^2^ Clinical Research Coordinating Team, Korea Institute of Oriental Medicine, Daejeon, Republic of Korea; ^3^ Department of Korean Medicine Ophthalmology and Otolaryngology and Dermatology, Daejeon Korean Medicine Hospital of Daejeon University, Daejeon, Republic of Korea; ^4^ Department of Korean Rehabilitation Medicine, Dongguk University Ilsan Oriental Hospital, Goyang, Republic of Korea

**Keywords:** atopic dermatitis, gastrointestinal microbiome, anorexia, herbal medicine, *Bojungikgi-tang*

## Abstract

**Introduction:**

Atopic dermatitis (AD) is often associated with gastrointestinal symptoms, including anorexia and alterations in the gut microbiome. A traditional East Asian herbal medicine *Bojungikgi-tang* (BJT; *Buzhongyiqi-tang* in Chinese, *Hochuekki-To* in Japanese) has been commonly used to manage AD and anorexia. This study aimed to evaluate the effects and safety of BJT in anorexic patients with AD and to investigate its therapeutic mechanism through gut microbiome analysis.

**Methods:**

The trial included 26 adults with AD and anorexia, randomized (1:1) into BJT or waiting list groups for 12 weeks, including 8 weeks of treatment and 4 weeks of follow-up. The severity of anorexia and AD was assessed using the Visual Analog Scale (VAS), SCORing of Atopic Dermatitis (SCORAD), and Dermatology Life Quality Index (DLQI). Serum cytokine concentrations were measured before and after treatment using a multiplex immunoassay. Fecal samples were collected before and after treatment, and 16S rRNA sequencing was performed to analyze the gut microbiota.

**Results:**

The BJT group showed a significant decrease in anorexia VAS scores compared to the waiting list group at 8 weeks. Only the BJT group showed significant improvements in SCORAD and DLQI scores compared to baseline, with effects maintained through follow-up. No serious adverse events related to BJT were reported. Among the serum cytokines, IL-1β significantly decreased only in the BJT group, while IL-4 and IL-17 significantly decreased in both groups, with a more pronounced reduction observed in the BJT group. After 8 weeks of BJT treatment, significant changes were observed in the gut microbiome, including alterations in the *Gemella* genus, *Gemmiger formicilis*, and *Blautia_uc* species.

**Conclusion:**

BJT may improve symptoms of anorexia and AD without serious adverse events, potentially through modifications in the gut microbiota.

**Clinical Trial Registration:**

The study protocol was registered at the Clinical Research Information Service (KCT0006784, https://cris.nih.go.kr/cris/search/detailSearch.do?seq=21298&search_page=L).

## 1 Introduction

Atopic dermatitis (AD) is a common chronic inflammatory skin disease marked by pruritus, eczema, dry skin, and itching (Kolb and Ferrer-Bruker, 2024). It affects 2.6% of the global population and imposes significant direct and indirect costs, creating a substantial socioeconomic burden (Tian et al., 2023; Urban et al., 2021; Chung and Simpson, 2019). AD can impact the body’s immune response, including the secretion of inflammatory cytokines, which may reduce appetite and alter intestinal microbiota composition (Lee et al., 2018). Additionally, gut microbiota disturbances can affect the immune system and skin barrier function, potentially leading to AD (Mao et al., 2024). Some patients with AD have food allergies, causing anxiety about eating and reducing appetite (Silverberg, 2019). Moreover, digestive system issues like poor digestion, anorexia, and leaky gut syndrome can lead to gastrointestinal symptoms and immune-related disorders such as AD (Caffarelli et al., 1998), suggesting a link between AD and anorexia.

Herbal medicine is widely used to manage AD and anorexia (Cai et al., 2022; Lee et al., 2022a). It can help treat and prevent diseases by regulating gut microbiome composition (An et al., 2019; Ko et al., 2024). *Bojungikgi-tang* (BJT; *Buzhongyiqi-tang* in Chinese, *Hochuekki-To* in Japanese) is a well-known herbal medicine comprising 10 herbs: *Panax ginseng* C. A. Meyer, *Atractylodes japonica* Koidzumi, *Astragalus membranaceus* Bunge, *Angelica gigas* Nakai, *Zizyphus jujuba* Miller var. inermis Rehder, *Bupleurum falcatum* Linné, *Citrus unshiu* Markovich, *Glycyrrhiza uralensis* Fischer, *Cimicifuga heracleifolia* Komarov, and *Zingiber officinale* Roscoe. It was first recorded in Dongwon Ten Medical Books. It is known for tonifying the middle energizer (the upper abdominal cavity, i.e., the portion between the diaphragm and the umbilicus housing the spleen, stomach, liver, and gallbladder) and strengthening qi, treating symptoms of qi deficiency such as fever, sweating, and lethargy (You et al., 2009). BJT has been used to treat AD and anorexia in clinical settings and research (Kobayashi et al., 2010; Cho et al., 2023). In AD mouse models, BJT alleviated dermatitis symptoms and reduced blood immunoglobulin E (IgE) levels, demonstrating immunomodulatory effects (Ahn et al., 2023; Kobayashi et al., 2003; Suzuki et al., 1999). However, previous studies on BJT’s efficacy in AD only targeted patients with qi deficiency and did not use internationally accepted tools like SCORing of Atopic Dermatitis (SCORAD) for symptom assessment, limiting their interpretation (Kobayashi et al., 2010). Additionally, no studies have examined BJT’s effect on gut microbiome changes, which are closely related to AD.

Therefore, in this study, we aimed to explore BJT’s effects and safety in anorexia patients with AD and the potential for future large-scale clinical trials. In addition, we examined BJT’s therapeutic mechanism for AD through gut microbiome analysis.

## 2 Methods

### 2.1 Trial design and eligibility criteria

This randomized, waiting list-controlled, assessor-blinded, parallel-design clinical trial was conducted at Daejeon Korean Medicine Hospital of Daejeon University (Daejeon, Republic of Korea) from December 2021 to December 2023. The trial lasted 12 weeks, including 8 weeks of BJT administration or waiting and 4 weeks of follow-up, with visits at 4-week intervals. The full schedule is in Supplementary Table S1.

This study was approved by the institutional review board of Daejeon Korean Medicine Hospital of Daejeon University (DJDSKH-21-DR-18). All participants gave written informed consent after receiving a detailed explanation of the study. The study protocol was registered at the Clinical Research Information Service (KCT0006784) and published previously (Lee et al., 2022b).

Participants were adults aged 19–65 years with anorexia severity ≥40 points on a 0–100 mm visual analog scale (VAS), diagnosed with AD per Hanifin & Rajka criteria (Hanifin and Rajka, 1980), and had mild to moderate AD with a SCORAD score of 15–50 points (Schallreuter et al., 1993). Exclusion criteria included underlying diseases causing anorexia, rapid weight loss of 10% or more within the past 6 months, use of appetite stimulants, health functional foods, or Korean medicine treatments for anorexia within 2 weeks. Also excluded were those with severe skin diseases other than AD, use of systemic immunosuppressants, oral antibiotics, oral corticosteroids, or systemic photochemotherapy within 4 weeks, use of topical immunomodulators, topical antibiotics, topical corticosteroids, or antihistamines within 1 week, or use of health functional foods or Korean medicine treatments for AD within 2 weeks. Detailed criteria are in the protocol paper (Lee et al., 2022b). Eligible participants were randomly assigned 1:1 to BJT or waiting list groups. This was the first trial to evaluate BJT’s effectiveness in participants with anorexia and AD. No prior studies were available for sample size calculation. As a preliminary study, we planned to recruit 40 participants, 20 per group, assuming a minimum effect size of 0.30 and considering a 20% dropout rate (Cocks and Torgerson, 2013).

### 2.2 BJT preparation and chemical component analysis

The BJT used in the trial was a fine granule of yellowish-brown to brown color manufactured by Kracie Pharma Korea Co., Ltd. (Seoul, Republic of Korea) in compliance with Korean good manufacturing practice standards (batch number: K262002). The BJT group took 7.5 g of BJT daily, divided into two doses of 3.75 g each, before or between meals for 8 weeks, as approved by the Korean Ministry of Food and Drug Safety (KMFDS). The daily active ingredient was 6,400 mg of BJT extracts, consisting of *P. ginseng* C. A. Meyer 4.0 g, *A. japonica* Koidzumi 4.0 g, *A. membranaceus* Bunge 4.0 g, *A. gigas* Nakai 3.0 g, *Z. jujuba* Miller var. inermis Rehder 2.0 g, *B. falcatum* Linné 2.0 g, *C. unshiu* Markovich 2.0 g, *G. uralensis* Fischer 1.5 g, *C. heracleifolia* Komarov 1.0 g, and *Z. officinale* Roscoe 0.5 g. The supplied products satisfied appropriate quality control standards regarding appearance, purity, formulation uniformity, particle size, content, and microbial limits. The waiting list group received 2 weeks of BJT as compensation after the 12-week trial period.

The chemical profiling of BJT to identify the chemical composition was performed using ultra-performance liquid chromatography quadrupole exactive (QE) Orbitrap mass spectrometry (UPLC/QE Orbitrap MS, Thermo Fisher Scientific, Waltham, MA, United States) system equipped with heated electrospray ionization source. The dried powder of BJT was dissolved in 50% MeOH solution at a concentration of 10 mg/mL. The reveled signal was separated using an Acquity BEH C18 column (100 × 2.1 mm, 1.7 µm, Waters Corp., Milford, MA, United States), maintained at 40°C on a Dionex UltiMate 3000 binary gradient system (Thermo Fisher Scientific). The mobile phase consisted of water containing 0.1% (v/v) formic acid (solvent A) and acetonitrile (solvent B). The flow rate was set to 0.25 mL/min for the total 27 min run time and the injection volume of BJT solution was 2 μL. The linear gradient of UPLC was as follows: 3% B for 1 min, 3%–15% B from 1 min to 2 min, 15%–50% B from 2 min to 13 min, 100% B from 13 to 20 min, and then equilibrate until 27 min. The mass spectrometer was operated in positive ionization mode based on on Full MS and data-dependent MS2 scan mode with a mass range of 100–1500 m/z. The acquired naked data were processed using MS-Dial (Ver 4.90, http://prime.psc.riken.jp/) and also identified chemical components of the BJT based on the retention time, m/z of precursor, and the MS fragment pattern using in all publicly available mass spectral databases obtained from RIKEN (http://prime.psc.riken.jp/Metabolomics_Software/MS-DIAL/).

All participants were provided with a topical corticosteroid (Lidomax Cream 0.15%; Prednisolone Valeroacetate 1.5 mg/g) as a rescue medication and instructed to apply an amount equivalent to one index fingertip unit to a lesion the size of two adult palms. Participants recorded the date, site, and amount of rescue medication used in a diary. The investigator measured the remaining amount of topical corticosteroid in both groups using a precision scale. The use of appetite stimulants, antihistamines, oral antibiotics, corticosteroids (other than the provided rescue medication), immunosuppressants, systemic photochemotherapy, and Korean medicine treatments and health functional foods for managing anorexia and AD was prohibited during the trial.

### 2.3 Outcomes

Participants’ demographic information, smoking, drinking, caffeine intake, exercise, and allergy history were collected during the first visit. Additionally, based on KMFDS clinical trial guidelines for herbal medicines, the pattern identification of AD was investigated (Korea Ministry of Food and Drug Safety, 2009).

To evaluate effectiveness, anorexia intensity was assessed using VAS (0–100 mm) as the primary outcome. Secondary outcomes included body weight, body fat percentage, body fat mass, skeletal muscle mass, SCORAD, Validated Investigator Global Assessment Scale for Atopic Dermatitis (vIGA-AD), Dermatology Life Quality Index (DLQI), EuroQoL 5 Dimension 5 Level (EQ-5D-5L), frequency and amount of topical corticosteroid (rescue medication) use, deficiency and excess scores based on the Deficiency and Excess Pattern Identification Questionnaire (DEPIQ) (Baek et al., 2020), and blood immune biomarkers, such as total IgE, eosinophil count, and cytokines. The concentrations of cytokines were measured using a multiplex immune-bead assay (Bio-Plex Pro Human Cytokine Grp 1 Panel 27-plex, #500KCAF0Y, BioRad). For safety, liver and kidney function tests were performed before and after BJT administration, and adverse events were monitored throughout the trial.

To assess feasibility, recruitment rate (the percentage of enrolled participants relative to the total number of screened participants), adherence rate (the percentage of participants who took 70% of the total investigational products relative to the total number of enrolled participants in BJT group), medication compliance in BJT group, and completion rate (the percentage of participants who completed the clinical trial without dropping out relative to the total number of enrolled participants) were calculated. Participants collected feces using pre-distributed kits for gut microbiome analysis before and after BJT administration. Outcomes were collected at screening (Visit 1), baseline (Visit 2), and weeks 4 (Visit 3), 8 (Visit 4), and 12 (Visit 5). Fecal samples were collected at baseline (Visit 2) and week 8 (Visit 4).

### 2.4 Randomization and blinding

The random allocation sequence was generated by an independent medical statistician using SAS^®^ Version 9.4 (SAS Institute Inc., Cary, NC) with a block randomization method (block size of 4) without stratification. Group assignments were placed in opaque, sealed envelopes and stored in a double-locked cabinet. If a participant met all eligibility criteria and was enrolled, the investigator opened the envelopes in order in front of the participant and assigned the participant to one of the two groups. Blinding participants and investigators was impossible due to the trial design, comparing BJT and the waiting list. However, outcome assessors were blinded to minimize bias.

### 2.5 Gut microbiome analysis

Total deoxyribonucleic acid (DNA) was extracted from fecal samples using the Maxwell RSC PureFood GMO and Authentication Kit (Promega) following the manufacturer’s instructions. Polymerase chain reaction (PCR) amplification targeted the V3 to V4 regions of the 16S rRNA gene using fusion primers. The primers for bacterial amplification were 341F (5′-AATGATACGGCGACCACCGAGATCTACAC-XXXXXXXX-TCGTCGGCAGCGTCAGATGTGTATAAGAGACAG-CCTACGGGNGGCWGCAG-3′; underlined sequence indicates the target region primer) and 805R (5′- CAAGCAGAAGACGGCATACGAGAT-XXXXXXXX-GTCTCGTGGGCTCGG-AGATGTGTATAAGAGACAG-GACTACHVGGGTATCTAATCC-3′). Fusion primers included P5 (P7) graft binding, i5 (i7) index, Nextera consensus, sequencing adaptor, and target region sequence. PCR conditions were as follows: initial denaturation at 95°C for 3 min, 25 cycles of denaturation at 95°C for 30 s, primer annealing at 55°C for 30 s, extension at 72°C for 30 s, and final elongation at 72°C for 5 min. PCR products were confirmed using 1% agarose gel electrophoresis and visualized under a Gel Doc system (BioRad, Hercules, CA, United States). Amplified products were purified using CleanPCR (CleanNA). Equal concentrations of purified products were pooled, and short fragments (non-target products) were removed with CleanPCR (CleanNA). Product quality and size were assessed on a Bioanalyzer 2100 (Agilent, Palo Alto, CA, United States) using a DNA 7500 chip. Mixed amplicons were pooled, and sequencing was performed at CJ Bioscience, Inc. (Seoul, Korea) using the Illumina MiSeq Sequencing system (Illumina, United States) following the manufacturer’s instructions.

The 16S rRNA gene sequences were identified using the Microbiome Taxonomic Profiling (MTP) pipeline from CJ Bioscience, Inc. (Yoon et al., 2017). Quality control of raw data followed CJ Bioscience’s in-house protocols (Lee et al., 2017; Parks et al., 2015). Taxonomic assignments were made using the USEARCH tool, which calculates sequence similarity against reads in the EzBioCloud database (https://www.ezbiocloud.net) (Yoon et al., 2017). Bacterial operational taxonomic units (OTUs) were identified using UCLUST, clustering the 16S rRNA sequences with a ≥97% identity threshold for taxonomic profiling. Diversity calculations and biomarker discovery were conducted using in-house programs at CJ Bioscience, Inc., with all analyses performed on the EzBioCloud 16S-based MTP platform.

### 2.6 Statistical analysis

All statistical analyses were conducted using two-sided tests with baseline as a covariate, at a 5% significance level, using SAS^®^ Version 9.4 (SAS Institute. Inc., Cary, NC). Following the intention-to-treat principle, data from all participants with at least one post-baseline measurement were included in the full analysis set (FAS). Depending on normality, continuous data were analyzed using the independent t-test or Wilcoxon rank sum test for between-group differences, and categorical data were analyzed using the Chi-square test or Fisher’s exact test. Within-group changes were analyzed using paired t-test or Wilcoxon signed rank test for continuous variables and Chi-square test or Fisher’s exact test for categorical variables. Continuous data were presented as means and 95% confidence intervals, and categorical data as frequencies and percentages. Missing values were replaced by multiple imputations.

## 3 Results

### 3.1 Chemical components of BJT

**FIGURE 1:**
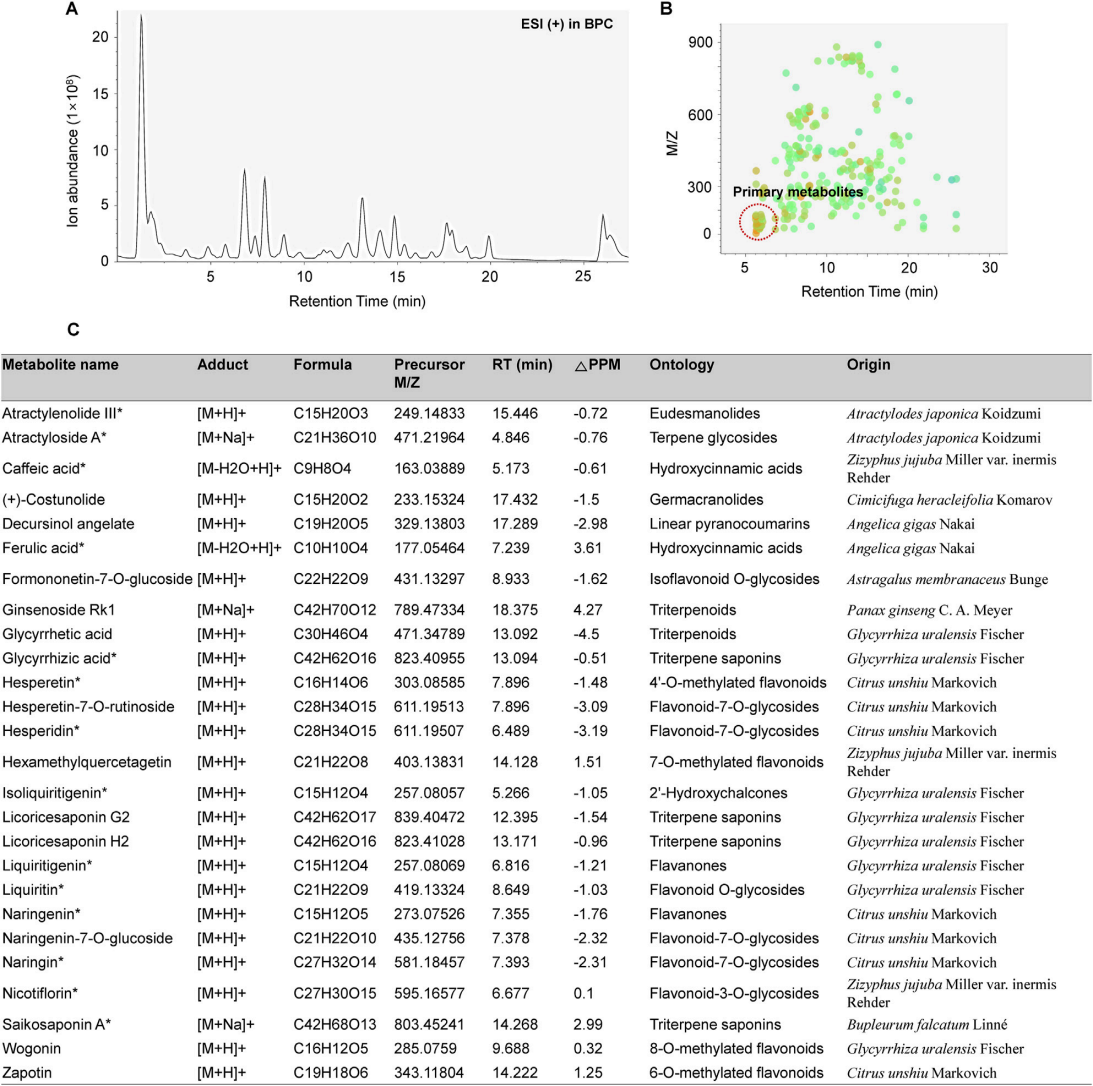
Chemical profiling of BJT. **(A)** Base peak chromatograms, **(B)** Peak spot graph representing identified metabolites, and **(C)** List of major chemical components.

The major chemical components in BJT were identified based on UPLC/QE Orbitrap MS analysis according to their relative retention time, m/z of precursor, and MS/MS fragments. A total of 1892 naked features were detected, and 203 metabolites were putatively matched with reference peaks of primary and secondary metabolites (Figures 1A,B). Among them, we determined 26 major compounds, including astragaloside IV, atractylenolide III, atractyloside A, caffeic acid, costunolide, decursinol angelate, ferulic acid, formonetic-7-O-glucoside, ginsenoside Rk1, glycyrrhetic acid, glycyrrhizic acid, hesperetin, hesperetin-7-O-rutinoside, hesperidin, hexamethylquercetagetin, isoliquiritigenin, licoricesaponin G2, licoricesaponin H2, liquiritigenin, liquiritin, naringenin, naringenin-7-O-glucoside, naringin, nicotiflorin, saikosaponin A, wogonin, and zapotin which were derived from each single extract comprising BJT. These major compounds were putatively annotated based on spectral matching at Metabolomics Standards Initiative (MSI) Level 2. Subsequently, 14 compounds (atractylenolide III, atractyloside A, caffeic acid, ferulic acid, glycyrrhizic acid, hesperetin, hesperidin, isoliquiritigenin, liquiritigenin, liquiritin, naringenin, naringin, nicotiflorin, and saikosaponin A) were further confirmed through direct comparison with authentic reference standards, thereby satisfying the criteria for MSI Level 1. No major undesirable or toxic compounds were detected under the applied analytical conditions (Figure 1C).

### 3.2 Participant flow and general characteristics

**FIGURE 2:**
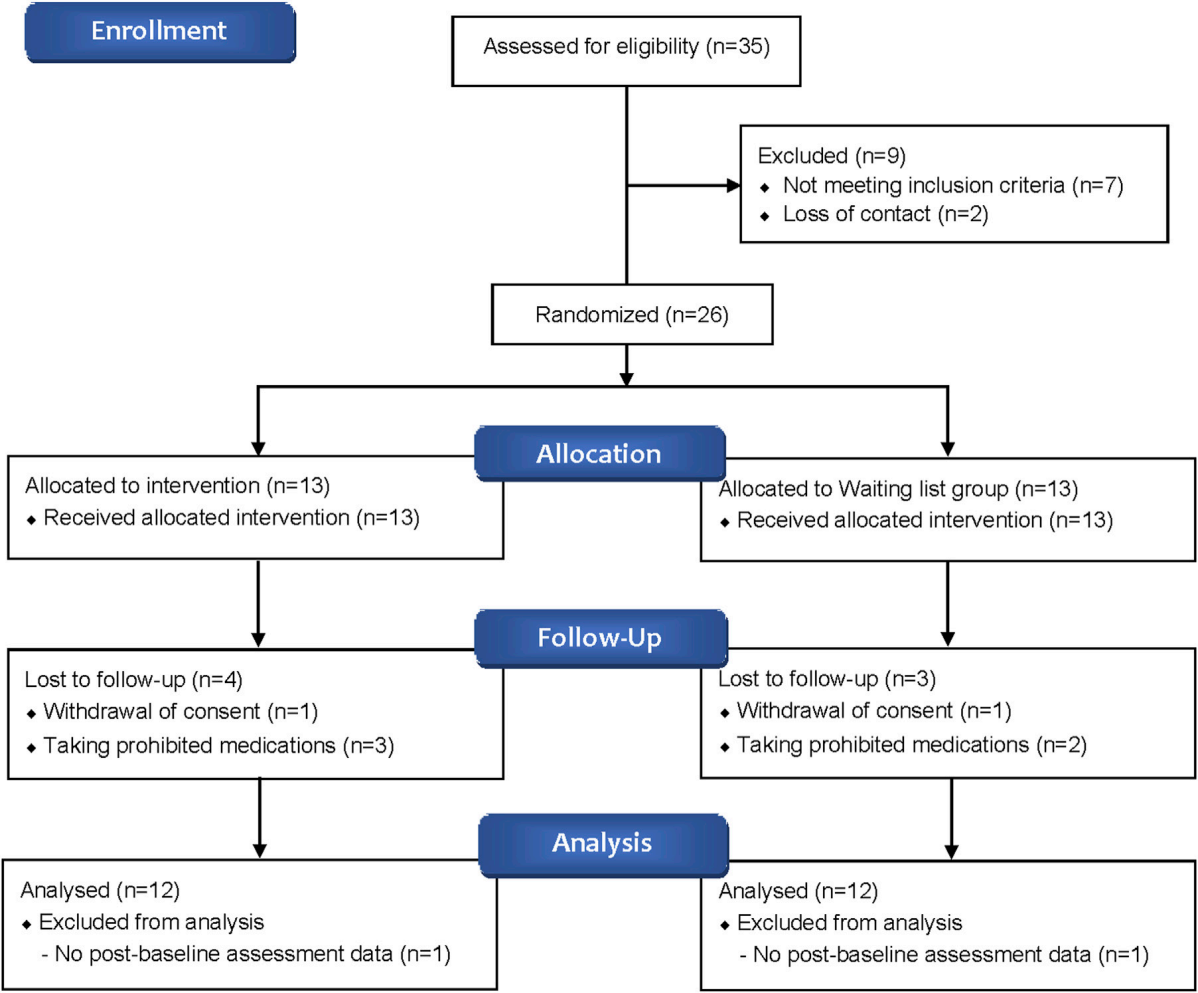
Flow diagram of study participants.

A total of 35 participants were screened, with 26 ultimately enrolled—13 in the BJT group and 13 in the waiting list group. Four participants from the BJT group and three from the waiting list group withdrew due to consent withdrawal or the use of prohibited medications. Consequently, data from 24 participants who met the FAS criteria were included in the analysis (Figure 2). There were no significant differences between the two groups in terms of baseline clinical characteristics, vital signs, and history of allergic diseases (Table 1).

**TABLE 1:** General characteristics of participants.

Characteristics	Bojungikgi-tang group (n = 12)	Waiting list group (n = 12)	*p*-value
Sex (Male/Female)	8 (66.67%)/4 (33.33%)	4 (33.33%)/8 (66.67%)	0.1025
Age (year)	28.83 (25.43, 32.24)	32.58 (23.37, 41.80)	0.4151
Smoking (Yes/No)†	5 (41.67%)/7 (58.33%)	3 (25.0%)/9 (75.0%)	0.6668
Drinking (Yes/No)†	9 (75.0%)/3 (25.0%)	7 (58.33%)/5 (41.67%)	0.6668
Caffeine intake (Yes/No)†	9 (75.0%)/3 (25.0%)	9 (75.0%)/3 (25.0%)	0.9999
Exercise (Yes/No)†	7 (58.33%)/5 (41.67%)	8 (66.67%)/4 (33.33%)	0.9999
BMI (kg/m^2^)	23.29 (21.47, 25.11)	23.32 (20.79, 25.84)	0.9861
Systolic blood pressure (mmHg)	125.0 (111.96, 138.04)	118.67 (109.99, 127.35)	0.3832
Diastolic blood pressure (mmHg)	70.25 (61.36, 79.14)	68.75 (59.77, 77.73)	0.7963
Pulse rate (bpm)	81.33 (75.50, 87.17)	75.25 (69.41, 81.09)	0.1191
Body temperature (°C)	36.54 (36.47, 36.62)	36.48 (36.35, 36.61)	0.3381
Anorexia morbidity period (year)	4.92 (1.44, 8.39)	4.33 (0.53, 8.14)	0.8056
AD morbidity period (year)	21.33 (17.40, 25.20)	16.08 (11.04, 21.13)	0.0826
Number of HRC main criteria met	3.67 (3.35, 3.98)	3.50 (3.17, 3.83)	0.4298
Number of HRC minor criteria met	8.75 (7.06, 10.45)	7.08 (5.49, 8.67)	0.1287
History of allergic disease			
Food allergy†	5 (41.67%)	5 (41.67%)	0.9999
Asthma†	1 (8.33%)	1 (8.33%)	0.9999
Allergic rhinitis†	8 (66.67%)	10 (83.33%)	0.6404
Allergic conjunctivitis†	4 (33.33%)	3 (25.0%)	0.9999
Drug allergy†	2 (16.67%)	3 (25.0%)	0.9999
Others†	2 (16.67%)	2 (16.67%)	0.9999
AD pattern identification†			0.1999
Excess pattern	4 (33.33%)	3 (25.0%)	
Deficiency pattern	8 (66.67%)	6 (50.0%)	
Not applicable	0 (0.0%)	3 (25.0%)	

^†^ Fisher`s exact test.

AD, atopic dermatitis; BMI, body mass index; HRC, Hanifin & Rajka criteria.

### 3.3 Improvement in anorexia following BJT administration

**FIGURE 3:**
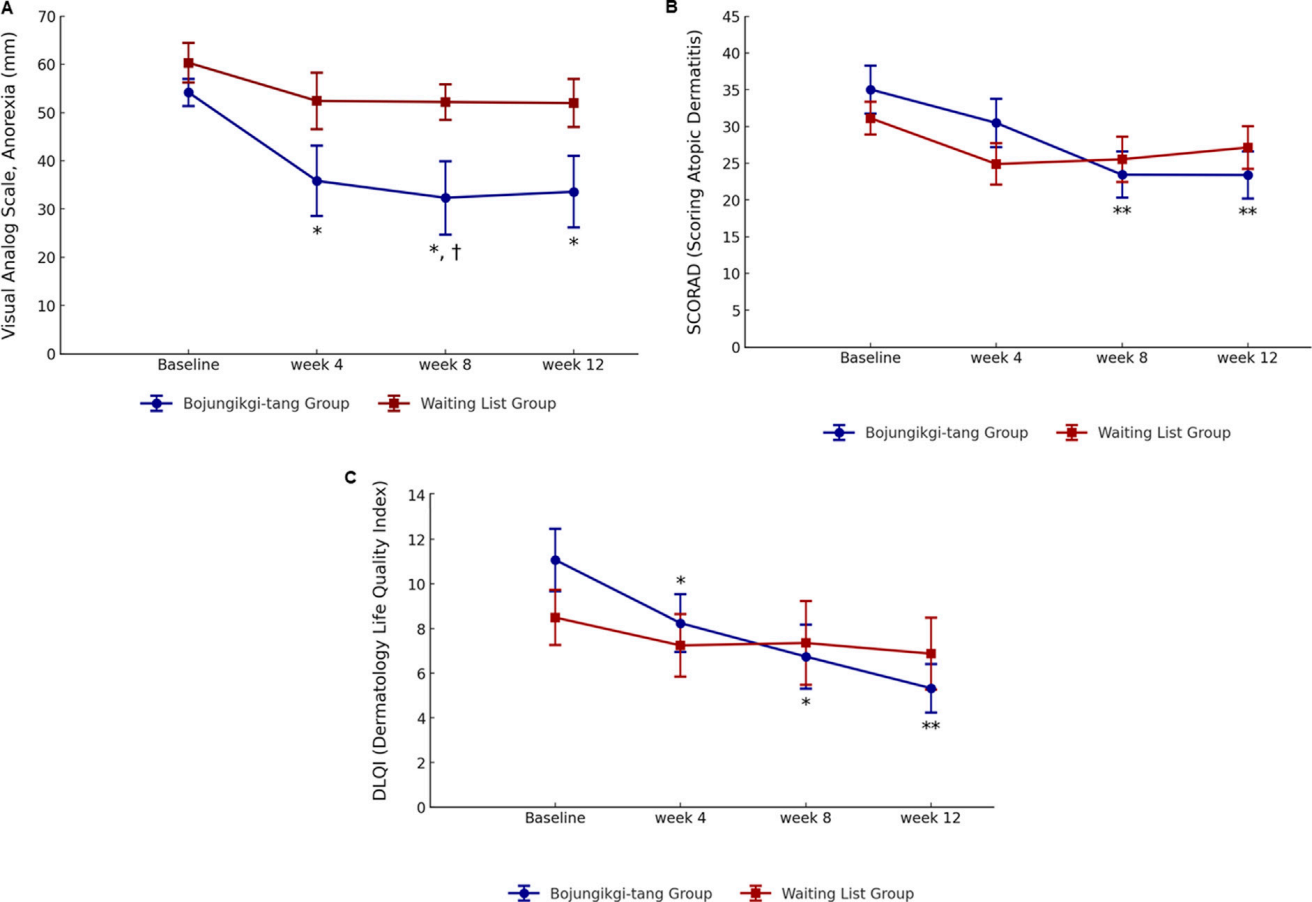
Changes in anorexia and atopic dermatitis symptoms. **(A)** Anorexia visual analog scale, **(B)** SCORing of Atopic Dermatitis, and **(C)** Dermatology Life Quality Index ∗, statistically significant differences (*p* < 0.05) compared to baseline; ∗∗, statistically significant differences (*p* < 0.01) compared to baseline; †, statistically significant differences (*p* < 0.05) between the *Bojungikgi-tang* group and the waiting list group.

The VAS score for anorexia intensity significantly decreased in the BJT group at week 8 compared to the waiting list group (*p* = 0.0347). Additionally, in the BJT group, the anorexia VAS score significantly decreased from baseline at weeks 4 (*p* = 0.0219) and 8 (*p* = 0.0155) of BJT administration, with the effect persisting until week 12 (*p* = 0.0157), 4 weeks after discontinuation. There was no significant within-group difference in the waiting list group in the anorexia VAS score (Table 2; Figure 3A). The two groups showed no significant differences in body weight, body mass index (BMI), or body fat percentage. After 8 weeks, body fat mass increased in the BJT group compared to the waiting list group (*p* = 0.0470), while skeletal muscle mass decreased (*p* = 0.0142) (Supplementary Table S2).

**TABLE 2:** Changes in anorexia symptom.

Visual analog scale	Bojungikgi-tang group (n = 12)	Waiting list group (n = 12)	Mean difference	*p*-value
Baseline	54.17 (47.93, 60.41)	60.33 (51.27, 69.40)	−6.17 (−16.54, 4.20)	0.2305
week 4	35.83 (19.79, 51.88)	52.42 (39.48, 65.35)	−11.97 (−30.86, 6.92)	0.2018
Difference	−18.33 (−33.47, −3.20)	−7.92 (−19.56, 3.73)		
*p*-value	0.0219*	0.1626		
week 8	32.33 (15.56, 49.10)	52.19 (44.11, 60.26)	−20.19 (−38.80, −1.59)	0.0347*
Difference	−21.83 (−38.63, −5.03)	−8.15 (−22.25, 5.95)		
*p*-value	0.0155*	0.2296		
week 12	33.58 (17.30, 49.84)	51.978 (41.11, 62.85)	−16.66 (−35.97, 2.65)	0.0873
Difference	−20.59 (−36.48, −4.70)	−8.36 (−21.87, 5.16)		
*p*-value	0.0157*	0.2009		

Values are means (95% confidence intervals). *Bold values mean significant differences.

### 3.4 Improvement in AD following BJT administration

Although there was no difference between the two groups, the SCORAD total score significantly decreased from baseline during the clinical trial period only in the BJT group (week 8, *p* = 0.0078; week 12, *p* = 0.0089), with no change observed in the waiting list group (Table 3; Figure 3B). While there was no significant difference within the groups in the objective symptoms score of the SCORAD index, only the BJT group showed a tendency for continuous improvement until the follow-up period after 8 weeks of administration. The subjective symptoms scores of the SCORAD index significantly improved at weeks 8 (*p* = 0.0082) and 12 (*p* = 0.0309) only in the BJT group, with particular improvement in pruritus symptoms. Additionally, vIGA-AD scores were lower in the waiting list group compared to the BJT group at week 4 (*p* = 0.0194); however, there was no significant difference between groups at weeks 8 and 12 (Supplementary Table S3).

**TABLE 3:** Changes in atopic dermatitis symptom.

Atopic dermatitis outcomes	Bojungikgi-tang group (n = 12)	Waiting list group (n = 12)	Mean difference	*p*-value
SCORAD Index				
Baseline	35.04 (27.87, 42.20)	31.13 (26.27, 35.98)	3.91 (−4.25, 12.06)	0.3313
week 4	30.49 (23.21, 37.78)	24.9 (18.73, 31.07)	3.38 (−4.72, 11.49)	0.3954
Difference	−4.54 (−10.73, 1.65)	−6.23 (−12.79, 0.33)		
*p*-value	0.1347	0.0607		
week 8	23.44 (16.54, 30.35)	25.54 (18.75, 32.34)	−3.41 (−12.55, 5.73)	0.4468
Difference	−11.59 (−19.46, −3.73)	−5.59 (−13.12, 1.95)		
*p*-value	0.0078*	0.1311		
week 12	23.40 (16.35, 30.44)	27.15 (20.72, 33.57)	−5.09 (−14.05, 3.86)	0.2498
Difference	−11.64 (−19.71, −3.57)	−3.98 (−10.99, 3.02)		
*p*-value	0.0089*	0.2369		
DLQI				
Baseline	11.08 (7.76, 14.41)	8.50 (5.78, 11.22)	2.58 (−1.46, 6.63)	0.1988
week 4	8.25 (5.14, 11.36)	7.25 (4.18, 10.32)	−0.59 (−4.09, 2.92)	0.7312
Difference	−2.83 (−5.06, −0.6)	−1.25 (−4.4, 1.9)		
*p*-value	0.0174*	0.401		
week 8	6.75 (3.32, 10.18)	7.36 (3.25, 11.48)	−2.37 (−6.89, 2.15)	0.2875
Difference	−4.33 (−7.47, −1.19)	−1.14 (−4.63, 2.36)		
*p*-value	0.0113*	0.4895		
week 12	5.33 (2.87, 7.78)	6.88 (3.31, 10.44)	−2.57 (−6.57, 1.43)	0.1963
Difference	−5.76 (−9.25, −2.26)	−1.62 (−4.85, 1.6)		
*p*-value	0.0040*	0.2919		
Number of topical corticosteroid applications				
week 0 to week 4	1.25 (−0.29, 2.79)	5.75 (1.91, 9.59)	−4.50 (−8.52, −0.48)	0.0308*
week 4 to week 8	0.92 (−0.18, 2.02)	5.80 (0.9, 10.70)	−4.88 (−9.84, 0.07)	0.0527
week 8 to week 12	4.09 (−1.25, 9.44)	5.50 (0.31, 10.69)	−1.41 (−8.39, 5.57)	0.6775
Total (week 0 to week 12)	6.46 (0.57, 12.34)	17.0 (2.35, 31.65)	−10.55 (−25.79, 4.70)	0.1576
Amount of topical corticosteroid applications (g)				
week 0 to week 4	0.47 (−0.19, 1.13)	1.48 (0.63, 2.33)	−1.01 (−2.01, −0.01)	0.0485*
week 4 to week 8	2.2 (−1.18, 5.57)	0.97 (0.15, 1.80)	1.22 (−2.21, 4.65)	0.4525
week 8 to week 12	1.65 (−0.38, 3.69)	0.56 (0.08, 1.05)	1.09 (−0.97, 3.15)	0.2693
Total (week 0 to week 12)	4.56 (0.36, 8.76)	3.29 (0.92, 5.67)	1.27 (−3.29, 5.83)	0.5622

Values are means (95% confidence intervals). *Bold values mean significant differences.

DLQI, dermatology life quality index; SCORAD, SCORing, of Atopic Dermatitis.

**FIGURE 4:**
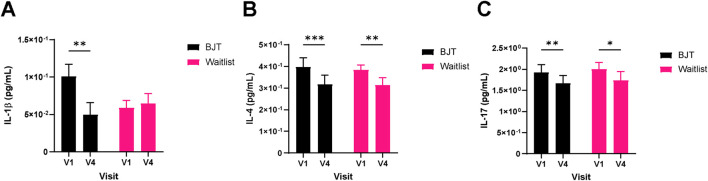
Changes in serum cytokine levels. **(A)** IL-1β, **(B)** IL-4, and **(C)** IL-17. Cytokine levels were measured in serum by a multiplex immune-bead assay. Abbreviations: BJT, *Bojungikgi-tang*; IL, interleukin. V1, Visit 1 (screening); V4, Visit 4 (week 8). Data are expressed as the mean ± SEM. Values below the detection limit were excluded from the analysis. *p < 0.05, **p < 0.01, ***p < 0.001; paired t-test.

Quality of life related to dermatitis, as assessed by DLQI, significantly improved only in the BJT group (week 4, *p* = 0.0174; week 8, *p* = 0.0113), with the effect persisting after the end of BJT administration (week 12, *p* = 0.0040) (Table 3; Figure 3C). There were no significant between- or within-group differences in overall health-related quality of life measured by EQ-5D-5L and blood immune biomarkers, including IgE and eosinophil (Supplementary Table S3). Among the serum cytokines, IL-1β (pro-inflammatory cytokine) significantly decreased only in the BJT group (*p* = 0.0057). IL-4 (Th2 cytokine) significantly decreased in both groups (BJT group, *p* = 0.0009; waiting list group, *p* = 0.0073), and IL-17 (Th17 cytokine) also significantly decreased in both groups (BJT group, *p* = 0.0079; waiting list group, *p* = 0.0114), but the reduction was more pronounced in the BJT group (Figure 4). Other cytokines, including those associated with AD, showed no significant changes after the administration of BJT (Supplementary Figure S1). The number and amount of topical corticosteroid use, a rescue medication, was significantly lower in the BJT group than in the waiting list group at week 4 (*p* = 0.0308 and 0.0485). However, groups did not differ in the total number of uses and amount used throughout the clinical trial period (Table 3).

### 3.5 Improvement in deficiency score after BJT administration

The deficiency score assessed by DEPIQ significantly decreased only in the BJT group after 8 weeks of administration (*p* = 0.0294). There was no significant difference in the excess score within or between groups (Table 4).

**TABLE 4:** Changes in deficiency and excess score.

Pattern identification	Bojungikgi-tang group (n = 12)	Waiting list group (n = 12)	Mean difference	*p*-value
Deficiency score				
Baseline	61.08 (52.58, 69.59)	60.33 (52.97, 67.69)	0.75 (−9.85, 11.35)	0.8847
week 8	51.67 (37.5, 65.84)	58.93 (51.75, 66.1)	−8 (−18.94, 2.93)	0.1429
Difference	−9.42 (−17.7, −1.13)	−1.41 (−9.1, 6.29)		
*p*-value	0.0294*	0.6951		
Excess score				
Baseline	49.42 (39.21, 59.63)	49.33 (39.12, 59.55)	0.08 (−13.53, 13.69)	0.9900
week 8	44.5 (33.19, 55.81)	50.19 (41.52, 58.87)	−5.76 (−14.52, 3.01)	0.1867
Difference	−4.92 (−11.36, 1.53)	0.86 (−6.39, 8.1)		
*p*-value	0.1214	0.7991		

Values are means (95% confidence intervals). *Bold values mean significant differences.

### 3.6 Gut microbiome changes after BJT administration

**FIGURE 5:**
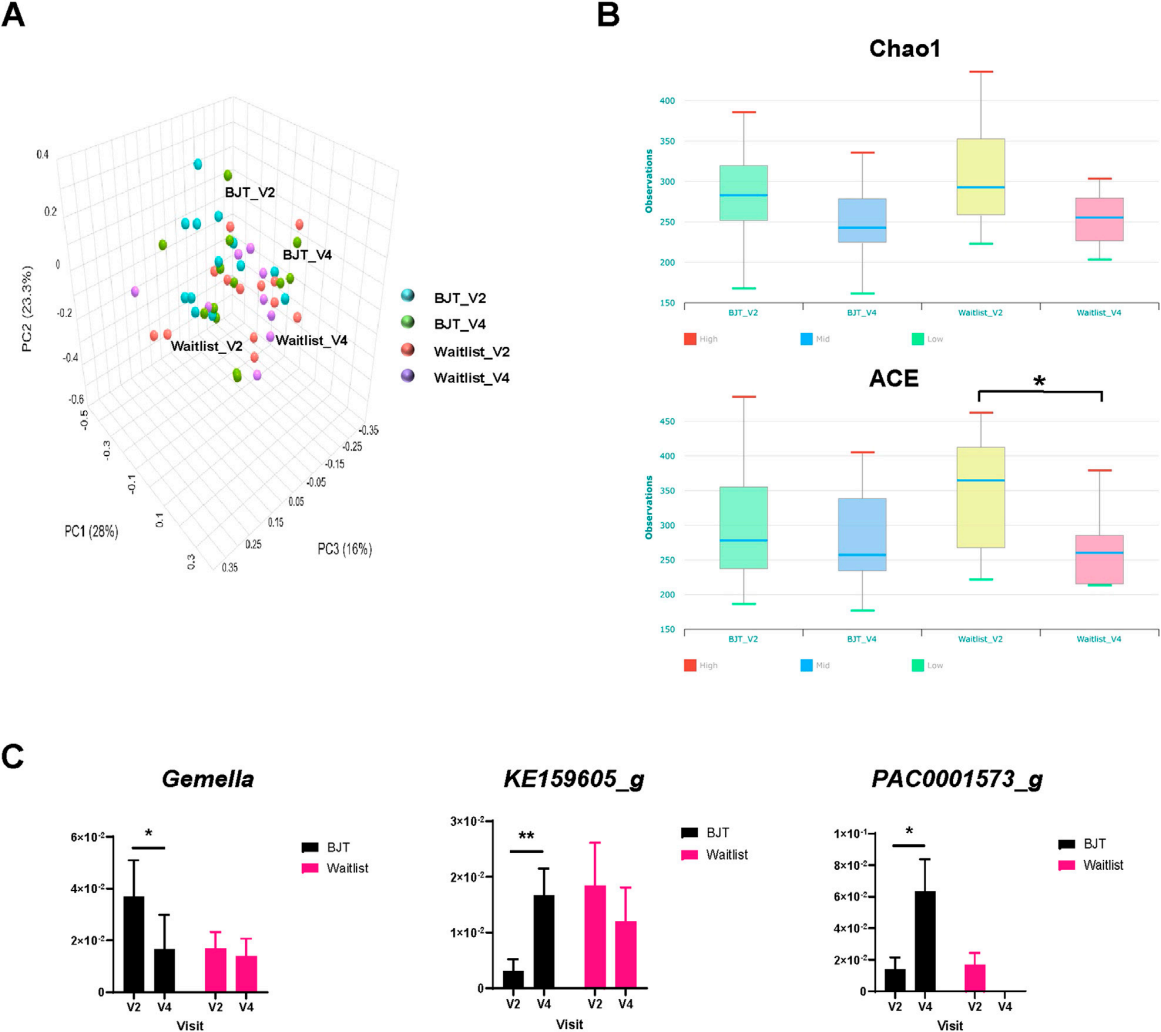
Gut microbiome composition. **(A)** Principal coordinates analysis of fecal microbiota composition, **(B)** changes in alpha diversity based on the Chao 1 and ACE index score, and **(C)** relative abundance of fecal microbiota significantly altered at the genus level Abbreviations: ACE, abundance-based Coverage Estimator; BJT, *Bojungikgi-tang*; V2, Visit 2 (baseline); V4, Visit 4 (week 8). Data are expressed as the mean ± SEM. Number of experiments: BJT_V2, n = 13; BJT_V4, n = 12; Waitlist_V2, n = 13, Waitlist_V4, n = 10. *, *p* < 0.05; **, *p* < 0.01; paired t-test.

The principal coordinates analysis (PCoA) of UniFrac distances between the groups revealed a distinct microbiome pattern (Figure 5A), although no significant differences were found. Notably, the alpha diversity index, specifically the Abundance-based Coverage Estimator (ACE), showed a significant decrease in the waiting list group after 8 weeks (Figure 5B). Additionally, in participants with a deficiency pattern of AD at baseline (Korea Ministry of Food and Drug Safety, 2009), the ACE index tended to be lower than those with an excess pattern, with a *p*-value of 0.073 (Supplementary Figure S2).

At the genus and species levels, three and eight taxa, respectively, showed significant differences between the two groups. At the genus level, *Gemella* significantly decreased after treatment in the BJT group, while *KE159605_g* and *PAC0001573_g* significantly increased (Figure 5C). Similarly, at the species level, the *Bifidobacterium catenulatum* group, *Gemmiger formicilis* group, *Parabacteroides merdae*, *PAC001396_s*, *PAC001597_s*, and *PAC002060_s* showed significant increases after treatment in the BJT group. At the same time, *Blautia_uc* and *DQ799511_s* exhibited significant reductions (Supplementary Figure S3).

### 3.7 Study feasibility

The recruitment rate was 74.29% (26/35) and the completion rate was 73.08% (19/26). The completion rates for the BJT and waiting list groups were 69.23% (9/13) and 76.92% (10/13), respectively. The adherence rate of the BJT group was 84.62% (11/13), primarily because two participants dropped out due to taking concomitant prohibited medications and withdrawing consent. The average 8-week medication compliance rate for BJT was 87.87%.

### 3.8 Safety analysis

There were no significant changes in blood test values, including those related to liver and kidney function, before and after BJT administration within the group (Supplementary Table S4). Adverse events occurred in 10 cases in the BJT group and five cases in the waiting list group, with no significant difference between the groups (*p* = 0.7602). One case of unrecovered fasting blood glucose elevation was noted in the BJT group; however, this participant had high blood glucose and HbA1c levels from the start of the clinical trial, making it unrelated to BJT. The remaining adverse events were resolved without special measures or through treatment. In the waiting list group, one case of elevated white blood cell and absolute neutrophil count values was observed, with the severity assessed as moderate. This was not related to the intervention and resolved spontaneously. The severity of the remaining adverse events was rated as mild. No serious adverse events occurred during the clinical trial period (Supplementary Table S5).

## 4 Discussion

This study evaluated the effectiveness, safety, and feasibility of BJT in participants with anorexia and AD. It explored the mechanism of BJT through gut microbiome analysis. After 8 weeks of BJT administration, anorexia VAS significantly decreased compared to baseline and the waiting list group. Meanwhile, body fat mass significantly increased, and skeletal muscle mass significantly decreased in the BJT group compared to the waiting list group after 8 weeks. This result might be due to the significant improvement in anorexia symptoms in the BJT group, leading to an increased amount of food consumed. Although we did not investigate the participants’ exercise volume and dietary composition during the trial period, if only the amount of food consumed had increased, body fat mass would have increased, resulting in corresponding changes. However, there was no statistical difference between groups in body fat percentage, body weight, and BMI after treatment. Although not statistically significant, there were considerable differences between groups in body fat mass and skeletal muscle mass at baseline, so careful interpretation is needed.

Regarding AD, although there were no differences between the two groups, after 8 weeks of treatment, only the BJT group showed significant decreases in the total SCORAD score, total subjective symptoms, and DLQI compared to baseline, with effects persisting for 4 weeks of follow-up. The minimal clinically important difference (MCID) of SCORAD was 8.7 points (Schram et al., 2012). In the BJT group, there was a decrease of 11.59 points at week 8 compared to baseline, indicating a clinically meaningful difference. Additionally, the MCID for inflammatory skin diseases of the DLQI was 4 points (Basra et al., 2015). The BJT group decreased by 4.33 points compared to baseline at week 8, which was also a clinically meaningful difference. Meanwhile, vIGA-AD significantly decreased in the waiting list group compared to the BJT group at week 4. The waiting list group used significantly more topical corticosteroids, a rescue medication, compared to the BJT group at week 4, which might have reduced AD symptoms such as erythema, similar to the previous study (Kobayashi et al., 2010). However, there was no difference between the two groups at week 8, and considering that the MCID of vIGA-AD was 1.0 (Simpson et al., 2022), the decrease of 0.33 points compared to baseline at week 4 within the waiting list group and the difference of 0.67 between the groups appeared to have no clinical significance. Although there was no significant difference in IgE and eosinophil count between the two groups after treatment, some cytokines, known to affect immune response regulation, skin barrier damage, pruritus, and inflammatory responses in AD patients (Fania et al., 2022), showed significant changes within the groups. In particular, IL-1β significantly decreased after treatment only in the BJT group. It is known that there is a direct correlation between increased IL-1β levels and AD severity, and that IL-1β affects the development, maintenance, and exacerbation of AD (Yeung et al., 2021). In addition, IL-4 is known to be one of the major causes of AD, contributing to the Th2 immune response, skin barrier damage, and increased pruritus (Chiricozzi et al., 2020). In our study, IL-4 significantly decreased in both groups after treatment, with a greater decrease observed in the BJT group. Furthermore, IL-17 significantly decreased in both groups after treatment, and the decrease was greater in the BJT group. IL-17 is a cytokine related to allergic inflammation, and the serum IL-17 level in AD patients tends to be higher than that in the healthy control group and showed a positive correlation with the SCORAD score (Hofmann et al., 2021). Based on the results, the treatment with BJT may appear to modulate several cytokines, including IL-1β, IL-4, and IL-17, which are known to play key roles in immune response regulation, skin barrier integrity, pruritus, and inflammation in AD patients, suggesting that BJT’s therapeutic mechanism may involve the suppression of pro-inflammatory cytokines and the restoration of immune balance.

The DEPIQ consists of 20 questions designed to assess deficiency and excess patterns, which are central to traditional Korean medicine (TKM) diagnostics (Baek et al., 2020; Jang et al., 2018). Deficiency patterns in TKM reflect weaknesses in vital substances (e.g., qi, blood, yin, or yang), often presenting as chronic fatigue, cold intolerance, or weak pulse (Jang et al., 2018). In contrast, excess patterns involve pathogenic factors (e.g., heat or dampness) and manifest as acute symptoms such as irritability or pain exacerbated by pressure (Jang et al., 2018). Although direct research linking DEPIQ scores to pattern identification in specific conditions such as AD is limited (Yun and Choi, 2012), the questionnaire’s alignment with the diagnostic principles of TKM supports its utility in clinical practice. Further investigation of the correlation between DEPIQ-derived deficiency/excess scores and AD pattern identification in TKM could enhance the tool’s applicability in both research and practice.

In our study, the deficiency scores measured by the DEPIQ were significantly decreased from baseline at week 8 in the BJT group only. BJT is a representative herbal medicine that tonifies the middle energizer (the upper abdominal cavity, i.e., the portion between the diaphragm and the umbilicus housing the spleen, stomach, liver, and gallbladder) and strengthens the qi, alleviating symptoms of qi deficiency such as fever, sweating, and sluggishness in TKM (You et al., 2009). Therefore, deficiency symptoms would significantly improve after taking BJT.

In our study, BJT influenced changes in the gut microbiome, which might have affected symptoms of anorexia and AD. The species richness of intestinal microbiota significantly decreased in week 8 compared to baseline in the waiting list group, while there was no significant difference in the BJT group. Additionally, compared to participants with excess patterns, those with deficiency patterns of AD tended to have lower species richness of gut microbiota. This might be explained by insufficient qi and compromised digestive function in deficiency patterns, which can lead to poor nutrient absorption and a reduced capacity to sustain microbial diversity. However, when BJT, primarily used for the treatment of deficiency symptoms, was administered, the diversity of fecal microbiota tended to be maintained compared to the baseline. Considering that the diversity of gut microbiota is lower in patients with AD compared to healthy individuals (Abrahamsson et al., 2012), it is meaningful that the results showed the maintenance of diversity only in the BJT group. Notably, after treatment, the *Gemella* genus was significantly reduced only in the BJT group. The microbiome of skin and fecal samples from patients with AD has been reported to have an increased abundance of the *Gemella* genus compared to healthy individuals (Łoś-Rycharska et al., 2021; Carrascosa-Carrillo et al., 2024; Chng et al., 2016). Additionally, the *G. formicilis* species significantly increased after treatment only in the BJT group. *Gemmiger formicilis* is known to produce butyrate, which plays an essential role in maintaining the health of the intestinal mucosa, suppressing inflammation, and regulating immune responses (Gossling and Moore, 1975; Canani et al., 2011). Furthermore, only in the BJT group *Blautia_uc* significantly decreased compared to the baseline. Although no study directly related *Blautia_uc* to AD at the species level, *Blautia* has been reported to be dominant in patients with AD (Wang et al., 2023). Therefore, BJT might induce changes in the gut microbiome, including *Blautia_uc*, thereby reducing the inflammatory response associated with AD. In this way, BJT affected the gut microbiome of anorexic participants with AD, which might have led to significant improvements in anorexia and AD symptoms in the BJT group.

No serious adverse events related to BJT occurred in this study, and no significant differences were found in blood test results before and after BJT administration. In summary, it is considered safe to administer BJT for 8 weeks to patients with anorexia and AD. The recruitment rate, adherence rate, and completion rate of this study were 74.29%, 84.62%, and 73.08%, respectively. The 8-week BJT medication compliance rate was high at 87.87%. The recruitment and medication compliance rates were at appropriate levels, indicating that the trial was feasible (Walters et al., 2017; Vrijens et al., 2024). However, there were quite a few dropouts, primarily due to taking concomitant prohibited medications such as antibiotics, antihistamines, and corticosteroids. This was because the trial was conducted during the coronavirus disease 2019 (COVID-19) period, and many participants were prescribed these medications for respiratory diseases. Additionally, due to funding issues caused by the prolonged COVID-19 pandemic, this trial did not reach its initial target number of participants. However, as it was the first trial to evaluate the effectiveness of BJT in participants with anorexia and AD without a calculated sample size, the impact on the research results is expected to be low. In future clinical trials, it may be possible to increase the completion rate by not prohibiting the concomitant use of relevant drugs for purposes other than AD and allowing use according to the researcher’s judgment. Additionally, the design of a future trial based on a larger sample size and with a long follow-up period would be useful for exploring the long-term effects of BJT on AD and anorexia with greater power.

A limitation of this trial is that the efficacy of BJT could not be confirmed due to the use of a waiting list control group. However, we tried to minimize the effects on the study results attributed to the inability to blind participants by blinding the outcome assessors. In particular, blinding of assessors is important for evaluating SCORAD objective symptoms scores, which showed a tendency to improve over the entire trial period only in the BJT group. Additionally, we confirmed for the first time the mechanism of BJT in patients with AD and anorexia through gut microbiome analysis.

## 5 Conclusion

After 8 weeks of administering the herbal medicine BJT, the intensity of anorexia, AD symptoms, and deficiency symptoms significantly improved in participants without serious adverse events. These improvements might be related to changes in the gut microbiome caused by BJT administration.

## Data Availability

The original contributions presented in the study are included in the article/Supplementary Material, further inquiries can be directed to the corresponding author.

## Abbreviations

AD, atopic dermatitis; BJT, Bojungikgi-tang; COVID-19, coronavirus disease 2019; DEPIQ, Deficiency and Excess Pattern Identification Questionnaire; DLQI, Dermatology Life Quality Index; DNA, deoxyribonucleic acid; EQ-5D-5L, EuroQoL 5 Dimension 5 Level; FAS, full analysis set; IgE, immunoglobulin E; KMFDS, Korean Ministry of Food and Drug Safety; MCID, minimal clinically important difference; MTP, Microbiome Taxonomic Profiling; OTU, operational taxonomic unit; PCR, Polymerase chain reaction; SCORAD, SCORing of Atopic Dermatitis; VAS, visual analog scale; vIGA-AD, Validated Investigator Global Assessment Scale for Atopic Dermatitis.
