# Supplementary material for: Effect of herbal medicine Bojungikgi-tang on gut microbiome and symptoms in anorexic patients with atopic dermatitis: a randomized controlled trial
Source: Front Pharmacol. 2025 Jun 3;16:1593477. doi: 10.3389/fphar.2025.1593477 (PMC12170585; doi:10.3389/fphar.2025.1593477)
Supplement: Supplementary file 1 [file Supplementaryfile1.docx]

Supplementary Table S1. Schedule of the clinical trial

|  | **Study period** | | | | |
| --- | --- | --- | --- | --- | --- |
|  | **Screening** | **Post-allocation** | | | **Follow-up** |
| **TIME POINT** | **~ 2 weeks (V1)** | **Week 0**  **(V2, baseline)** | **Week 4**  **(V3)** | **Week 8**  **(V4)** | **Week 12**  **(V5)** |
| ENROLMENT |  |  |  |  |  |
| Informed consent | ● |  |  |  |  |
| Eligibility screen | ● |  |  |  |  |
| Demographic characteristics and medical history taking | ● |  |  |  |  |
| Random allocation |  | ● |  |  |  |
| INTERVENTIONS |  |  |  |  |  |
| BJT administration^+^ |  | ←－- - - - - - - - - - - - - - - - - - - - - - - → | | |  |
| Distribution of topical corticosteroids (remedy) and usage diary |  | ● | ● | ● |  |
| ASSESSMENTS |  |  |  |  |  |
| Vital sign | ● | ● | ● | ● | ● |
| Physical examination | ● |  |  |  |  |
| Hanifin and Rajka criteria | ● |  |  |  |  |
| Atopic dermatitis pattern identification questionnaire |  | ● |  |  |  |
| Laboratory test | ● |  |  | ● |  |
| Fecal test (gut microbiome) |  | ● |  | ● |  |
| Anorexia VAS | ● |  | ● | ● | ● |
| Body weight, body fat percentage, body fat mass, skeletal muscle mass |  | ● | ● | ● | ● |
| SCORAD | ● |  | ● | ● | ● |
| vIGA-AD |  | ● | ● | ● | ● |
| DLQI |  | ● | ● | ● | ● |
| EQ-5D-5L |  | ● | ● | ● | ● |
| DEPIQ |  | ● |  | ● |  |
| 3-day food record |  | ● |  | ● |  |
| Adverse events |  | ● | ● | ● | ● |
| Compliance with investigational product^+^ |  |  | ● | ● |  |

^+^ Only in the BJT group.

BJT, Bojungikgi-tang; DEPIQ, deficiency and excess pattern identification questionnaire; DLQI, Dermatology Life Quality Index; EQ-5D-5L, EuroQoL 5 Dimension 5 Level; SCORAD, SCORing of Atopic Dermatitis; V1, Visit 1; V2, Visit 2; V3, Visit 3; V4, Visit 4; V5, Visit 5; VAS, visual analog scale; vIGA-AD, Validated Investigator Global Assessment scale for Atopic Dermatitis.

Supplementary Table S2. Changes in body measurement outcomes

| Body measurement | Bojungikgi-tang group (n=12) | Waiting list group (n=12) | Mean difference | *p*-value |
| --- | --- | --- | --- | --- |
| **Body weight (kg)** |  |  |  |  |
| Baseline | 69.40 (60.97, 77.83) | 61.73 (51.85, 71.62) | 7.67 (-4.57, 19.91) | 0.2074 |
| week 4 | 69.58 (60.97, 78.18) | 62.05 (51.76, 72.34) | -0.36 (-1.55, 0.82) | 0.5333 |
| Difference | 0.18 (-0.86, 1.21) | 0.32 (-0.36, 0.99) |  |  |
| *p*-value | 0.7174 | 0.3251 |  |  |
| week 8 | 69.39 (60.30, 78.48) | 61.44 (51.83, 71.06) | 0.21 (-1.57, 1.99) | 0.8082 |
| Difference | -0.01 (-1.46, 1.44) | -0.29 (-1.33, 0.75) |  |  |
| *p*-value | 0.9901 | 0.5503 |  |  |
| week 12 | 69.62 (60.72, 78.52) | 61.65 (51.64, 71.65) | 0.13 (-1.57, 1.83) | 0.8725 |
| Difference | 0.22 (-1.26, 1.71) | -0.09 (-0.96, 0.78) |  |  |
| *p*-value | 0.7503 | 0.8314 |  |  |
| **Body mass index (kg/m^2^)** |  |  |  |  |
| Baseline | 23.29 (21.47, 25.11) | 23.32 (20.79, 25.84) | -0.03 (-2.96, 2.91) | 0.9861 |
| week 4 | 23.38 (21.46, 25.31) | 23.39 (20.78, 26.01) | 0.02 (-0.37, 0.41) | 0.9269 |
| Difference | 0.09 (-0.24, 0.42) | 0.08 (-0.18, 0.33) |  |  |
| *p*-value | 0.5548 | 0.5341 |  |  |
| week 8 | 23.28 (21.20, 25.35) | 23.19 (20.84, 25.53) | 0.11 (-0.46, 0.69) | 0.6844 |
| Difference | -0.02 (-0.48, 0.45) | -0.13 (-0.50, 0.24) |  |  |
| *p*-value | 0.9389 | 0.4489 |  |  |
| week 12 | 23.38 (21.39, 25.38) | 23.27 (20.73, 25.81) | 0.14 (-0.43, 0.71) | 0.6190 |
| Difference | 0.09 (-0.41, 0.59) | -0.05 (-0.37, 0.27) |  |  |
| *p*-value | 0.6953 | 0.7540 |  |  |
| **Body fat percentage (%)** |  |  |  |  |
| Baseline | 25.88 (21.11, 30.64) | 32.30 (27.00, 37.60) | -6.43 (-13.14, 0.29) | 0.0598 |
| week 4 | 26.21 (21.32, 31.09) | 31.79 (26.81, 36.78) | 0.67 (-0.18, 1.51) | 0.1148 |
| Difference | 0.33 (-0.16, 0.83) | -0.51 (-1.17, 0.16) |  |  |
| *p*-value | 0.1649 | 0.1201 |  |  |
| week 8 | 27.18 (22.31, 32.04) | 31.76 (27.59, 35.93) | 1.02 (-0.61, 2.65) | 0.2062 |
| Difference | 1.30 (0.71, 1.89) | -0.54 (-2.24, 1.16) |  |  |
| *p*-value | **0.0005*** | 0.4975 |  |  |
| week 12 | 26.59 (21.73, 31.45) | 32.05 (27.62, 36.48) | 0.31 (-1.26, 1.88) | 0.6862 |
| Difference | 0.71 (-0.34, 1.77) | -0.25 (-1.53, 1.03) |  |  |
| *p*-value | 0.1641 | 0.6780 |  |  |
| **Body fat mass (kg)** |  |  |  |  |
| Baseline | 17.45 (14.93, 19.97) | 20.29 (15.03, 25.56) | -2.84 (-8.47, 2.79) | 0.3001 |
| week 4 | 17.66 (15.14, 20.17) | 19.96 (14.99, 24.92) | 0.40 (-0.03, 0.83) | 0.0670 |
| Difference | 0.21 (-0.15, 0.56) | -0.33 (-0.72, 0.05) |  |  |
| *p*-value | 0.2239 | 0.0814 |  |  |
| week 8 | 18.33 (15.57, 21.08) | 19.67 (15.31, 24.03) | 1.11 (0.02, 2.19) | **0.0470*** |
| Difference | 0.88 (0.19, 1.56) | -0.62 (-1.79, 0.55) |  |  |
| *p*-value | **0.0167*** | 0.2702 |  |  |
| week 12 | 18.02 (15.15, 20.89) | 20.06 (15.25, 24.86) | 0.61 (-0.71, 1.93) | 0.3444 |
| Difference | 0.57 (-0.41, 1.55) | -0.24 (-1.22, 0.75) |  |  |
| *p*-value | 0.2243 | 0.6082 |  |  |
| **Skeletal muscle mass (kg)** |  |  |  |  |
| Baseline | 28.93 (23.78, 34.09) | 22.57 (18.67, 26.46) | 6.37 (0.28, 12.46) | 0.0618 |
| week 4 | 28.89 (23.53, 34.25) | 22.91 (18.68, 27.14) | -0.71 (-1.43, 0.00) | 0.0505 |
| Difference | -0.04 (-0.65, 0.57) | 0.34 (-0.09, 0.77) |  |  |
| *p*-value | 0.8837 | 0.1091 |  |  |
| week 8 | 28.46 (23.14, 33.78) | 22.84 (18.75, 26.94) | -0.96 (-1.71, -0.21) | **0.0142*** |
| Difference | -0.48 (-1.09, 0.14) | 0.28 (-0.13, 0.68) |  |  |
| *p*-value | 0.1165 | 0.1585 |  |  |
| week 12 | 28.76 (23.53, 33.99) | 22.67 (18.67, 26.68) | -0.37 (-1.06, 0.32) | 0.2775 |
| Difference | -0.17 (-0.76, 0.42) | 0.11 (-0.17, 0.39) |  |  |
| *p*-value | 0.5414 | 0.4194 |  |  |

Values are means (95% confidence intervals). *Significant differences.

Supplementary Table S3. Changes in other outcomes related to atopic dermatitis, quality of life, and blood immune biomarkers

| Outcomes | Bojungikgi-tang group (n=12) | Waiting list group (n=12) | Mean difference | *p*-value |
| --- | --- | --- | --- | --- |
| **SCORAD - Extent of lesion** | | | | |
| Baseline | 6.30 (0.47, 12.14) | 3.35 (1.00, 5.70) | 2.95 (-3.16, 9.06) | 0.3190 |
| week 4 | 7.18 (0.38, 13.97) | 2.01 (1.18, 2.85) | 2.19 (-0.37, 4.75) | 0.0901 |
| Difference | 0.87 (-0.57, 2.32) | -1.34 (-3.49, 0.81) |  |  |
| *p*-value | 0.2114 | 0.1972 |  |  |
| week 8 | 4.14 (1.27, 7) | 2.55 (1.07, 4.04) | 0.46 (-1.68, 2.59) | 0.6620 |
| Difference | -2.17 (-5.66, 1.32) | -0.8 (-3.56, 1.96) |  |  |
| *p*-value | 0.1989 | 0.5367 |  |  |
| week 12 | 3.41 (1.89, 4.94) | 3.19 (0.19, 6.19) | 0.12 (-3.2, 3.44) | 0.9406 |
| Difference | -2.89 (-8.42, 2.65) | -0.16 (-4.35, 4.03) |  |  |
| *p*-value | 0.2750 | 0.9344 |  |  |
| **SCORAD - Intensity of lesion** | | | | |
| Baseline | 6.33 (4.68, 7.99) | 5.50 (4.37, 6.63) | 0.83 (-1.06, 2.72) | 0.3705 |
| week 4 | 5.92 (4.58, 7.26) | 4.25 (3.13, 5.37) | 1.26 (-0.16, 2.68) | 0.0800 |
| Difference | -0.42 (-1.55, 0.72) | -1.25 (-2.61, 0.11) |  |  |
| *p*-value | 0.4350 | 0.0677 |  |  |
| week 8 | 4.42 (2.63, 6.2) | 4.33 (3.1, 5.56) | -0.07 (-2.17, 2.03) | 0.9464 |
| Difference | -1.92 (-3.95, 0.12) | -1.17 (-2.93, 0.59) |  |  |
| *p*-value | 0.0625 | 0.1709 |  |  |
| week 12 | 4.56 (2.81, 6.31) | 4.78 (3.65, 5.91) | -0.52 (-2.45, 1.42) | 0.5848 |
| Difference | -1.78 (-3.65, 0.1) | -0.72 (-2.14, 0.69) |  |  |
| *p*-value | 0.0613 | 0.2858 |  |  |
| **SCORAD - Objective symptoms** | | | | |
| Baseline | 12.64 (6.57, 18.71) | 8.85 (5.88, 11.82) | 3.78 (-2.72, 10.29) | 0.2355 |
| week 4 | 13.09 (5.93, 20.26) | 6.26 (5.07, 7.46) | 3.83 (-3.22, 10.89) | 0.2569 |
| Difference | 0.46 (-1.72, 2.64) | -2.59 (-5.74, 0.57) |  |  |
| *p*-value | 0.6539 | 0.0983 |  |  |
| week 8 | 8.55 (4.74, 12.36) | 6.88 (4.65, 9.12) | 0.40 (-4.43, 5.23) | 0.8573 |
| Difference | -4.08 (-8.32, 0.15) | -1.97 (-5.91, 1.97) |  |  |
| *p*-value | 0.0573 | 0.2949 |  |  |
| week 12 | 7.97 (5.12, 10.82) | 7.97 (4.38, 11.56) | 0.92 (-4.37, 6.22) | 0.7084 |
| Difference | -4.66 (-10.72, 1.39) | -0.88 (-5.89, 4.12) |  |  |
| *p*-value | 0.118 | 0.7055 |  |  |
| **SCORAD - Pruritus** | | | | |
| Baseline | 6.76 (5.35, 8.17) | 6.09 (5.09, 7.10) | 0.67 (-0.96, 2.30) | 0.4057 |
| week 4 | 4.95 (3.28, 6.62) | 5.2 (3.28, 7.12) | -0.78 (-2.88, 1.32) | 0.4489 |
| Difference | -1.81 (-3.51, -0.1) | -0.89 (-2.23, 0.45) |  |  |
| *p*-value | **0.0395*** | 0.1708 |  |  |
| week 8 | 3.47 (2.16, 4.77) | 4.9 (3.04, 6.76) | -1.89 (-3.79, 0.01) | 0.0515 |
| Difference | -3.29 (-4.88, -1.7) | -1.2 (-2.42, 0.03) |  |  |
| *p*-value | **0.0008*** | 0.0554 |  |  |
| week 12 | 3.65 (1.68, 5.63) | 4.86 (3.14, 6.57) | -1.46 (-3.94, 1.02) | 0.2339 |
| Difference | -3.1 (-5.42, -0.79) | -1.23 (-2.69, 0.23) |  |  |
| *p*-value | **0.0132*** | 0.0899 |  |  |
| **SCORAD - Sleep disturbance** | | | | |
| Baseline | 4.85 (2.91, 6.79) | 5.12 (3.54, 6.69) | -0.27 (-2.62, 2.09) | 0.8164 |
| week 4 | 3.4 (1.6, 5.2) | 4.43 (2.76, 6.09) | -0.9 (-3.01, 1.2) | 0.3821 |
| Difference | -1.45 (-3.56, 0.66) | -0.69 (-2.16, 0.77) |  |  |
| *p*-value | 0.1577 | 0.3212 |  |  |
| week 8 | 3.69 (1.57, 5.82) | 4.66 (2.88, 6.44) | -0.83 (-3.22, 1.56) | 0.4768 |
| Difference | -1.16 (-3.25, 0.94) | -0.46 (-2.25, 1.34) |  |  |
| *p*-value | 0.2493 | 0.5865 |  |  |
| week 12 | 3.39 (1.14, 5.64) | 4.83 (3.36, 6.3) | -1.3 (-3.55, 0.95) | 0.2435 |
| Difference | -1.46 (-3.7, 0.78) | -0.29 (-1.58, 1.01) |  |  |
| *p*-value | 0.1781 | 0.6338 |  |  |
| **SCORAD - Subjective symptoms** | | | | |
| Baseline | 11.61 (8.92, 14.30) | 11.21 (8.99, 13.42) | 0.40 (-2.88, 3.68) | 0.8028 |
| week 4 | 8.35 (5.09, 11.61) | 9.63 (6.14, 13.11) | -1.59 (-5.4, 2.23) | 0.3968 |
| Difference | -3.26 (-6.64, 0.12) | -1.58 (-3.74, 0.58) |  |  |
| *p*-value | 0.0574 | 0.1350 |  |  |
| week 8 | 7.16 (4.07, 10.25) | 9.55 (6.05, 13.06) | -2.71 (-6.37, 0.95) | 0.1390 |
| Difference | -4.45 (-7.5, -1.4) | -1.66 (-4.02, 0.7) |  |  |
| *p*-value | **0.0082*** | 0.1508 |  |  |
| week 12 | 7.12 (3.04, 11.19) | 9.69 (6.79, 12.58) | -2.81 (-7.21, 1.59) | 0.1985 |
| Difference | -4.49 (-8.49, -0.49) | -1.52 (-4.09, 1.05) |  |  |
| *p*-value | **0.0309*** | 0.2192 |  |  |
| **vIGA-AD** | | | | |
| Baseline | 2.08 (1.51, 2.66) | 2.08 (1.66, 2.51) | 0.0 (-0.67, 0.67) | > 0.9999 |
| week 4 | 2.42 (1.91, 2.92) | 1.75 (1.27, 2.23) | 0.67 (0.12, 1.21) | **0.0194*** |
| Difference | 0.33 (-0.08, 0.75) | -0.33 (-0.83, 0.16) |  |  |
| *p*-value | 0.1039 | 0.1661 |  |  |
| week 8 | 2 (1.46, 2.54) | 1.81 (1.27, 2.35) | 0.19 (-0.54, 0.92) | 0.5936 |
| Difference | -0.08 (-0.82, 0.66) | -0.27 (-0.88, 0.33) |  |  |
| *p*-value | 0.8088 | 0.3394 |  |  |
| week 12 | 2.08 (1.34, 2.81) | 2.18 (1.65, 2.7) | -0.1 (-0.88, 0.69) | 0.8011 |
| Difference | 0 (-0.67, 0.66) | 0.09 (-0.48, 0.66) |  |  |
| *p*-value | 0.9878 | 0.7304 |  |  |
| **EQ-5D-5L** | | | | |
| Baseline | 0.84 (0.76, 0.92) | 0.84 (0.81, 0.88) | -0.003 (-0.086, 0.079) | 0.9349 |
| week 4 | 0.85 (0.79, 0.9) | 0.88 (0.81, 0.94) | -0.03 (-0.09, 0.04) | 0.3784 |
| Difference | 0 (-0.06, 0.07) | 0.03 (-0.02, 0.08) |  |  |
| *p*-value | 0.8866 | 0.1781 |  |  |
| week 8 | 0.85 (0.75, 0.95) | 0.89 (0.83, 0.95) | -0.04 (-0.12, 0.05) | 0.4010 |
| Difference | 0.01 (-0.06, 0.08) | 0.05 (-0.02, 0.11) |  |  |
| *p*-value | 0.7482 | 0.1267 |  |  |
| week 12 | 0.88 (0.82, 0.94) | 0.85 (0.77, 0.94) | 0.03 (-0.06, 0.12) | 0.4958 |
| Difference | 0.04 (-0.02, 0.1) | 0.01 (-0.07, 0.09) |  |  |
| *p*-value | 0.2019 | 0.8671 |  |  |
| **EQ-VAS** | | | | |
| Baseline | 64.08 (52.10, 76.07) | 68.50 (59.62, 77.38) | -4.42 (-18.47, 9.64) | 0.5213 |
| week 4 | 62.92 (48.77, 77.06) | 59.08 (48.67, 69.49) | 6.73 (-7.51, 20.97) | 0.3368 |
| Difference | -1.17 (-10.66, 8.33) | -9.42 (-21.62, 2.78) |  |  |
| *p*-value | 0.7919 | 0.1174 |  |  |
| week 8 | 56.42 (41.65, 71.19) | 64.18 (52.99, 75.37) | -6.63 (-24.33, 11.07) | 0.4449 |
| Difference | -7.67 (-23.97, 8.64) | -4.32 (-17.95, 9.31) |  |  |
| *p*-value | 0.3229 | 0.4997 |  |  |
| week 12 | 66.66 (56.34, 76.98) | 62.73 (54.47, 70.98) | 5.44 (-6.47, 17.35) | 0.3528 |
| Difference | 2.58 (-7.23, 12.39) | -5.77 (-17.99, 6.45) |  |  |
| *p*-value | 0.5742 | 0.3207 |  |  |
| **Immunoglobulin E** | | | | |
| Baseline (Screening) | 622.35 (-36.65, 1281.35) | 403.63 (-233.41, 1040.67) | 218.7 (-644.9, 1082.4) | 0.6047 |
| week 8 | 701.28 (-8.92, 1411.47) | 365.46 (-142.97, 873.88) | 131.86 (-43.17, 306.9) | 0.1321 |
| Difference | 78.93 (-57.75, 215.6) | -38.18 (-170.5, 94.11) |  |  |
| *p*-value | 0.2299 | 0.5383 |  |  |
| **Eosinophil** | | | | |
| Baseline (Screening) | 4.81 (1.73, 7.89) | 2.93 (2.08, 3.79) | 1.88 (-1.27, 5.02) | 0.2200 |
| week 8 | 3.48 (1.81, 5.14) | 2.96 (1.99, 3.93) | -0.45 (-1.46, 0.56) | 0.3621 |
| Difference | -1.33 (-3.03, 0.36) | 0.03 (-0.71, 0.77) |  |  |
| *p*-value | 0.1113 | 0.9331 |  |  |

Values are means (95% confidence intervals). *Significant differences.

EQ-5D-5L, EuroQoL 5 Dimension 5 Level; EQ-VAS, EuroQol Visual Analogue Scale; SCORAD, SCORing of Atopic Dermatitis; vIGA-AD, Validated Investigator Global Assessment scale for Atopic Dermatitis.

Supplementary Table S4. Changes in blood tests

| Blood tests | Bojungikgi-tang group (n=12) | Waiting list group (n=12) |
| --- | --- | --- |
| **WBC** |  |  |
| Baseline (Screening) | 5.46 (4.86, 6.06) | 5.74 (4.85, 6.63) |
| week 8 | 5.67 (4.84, 6.5) | 6.39 (4.21, 8.57) |
| Difference | 0.21 (-0.52, 0.93) | 0.68 (-0.63, 1.99) |
| *p-*value | 0.5395 | 0.2719 |
| **Neutrophils** |  |  |
| Baseline (Screening) | 64.10 (57.88, 70.32) | 66.14 (61.57, 70.71) |
| week 8 | 63.08 (58.99, 67.18) | 65.69 (59.81, 71.57) |
| Difference | -1.02 (-5.81, 3.77) | 0.52 (-2.41, 3.45) |
| *p-*value | 0.6495 | 0.6975 |
| **Lymphocyte** |  |  |
| Baseline (Screening) | 23.16 (19.79, 26.53) | 22.95 (18.89, 27.01) |
| week 8 | 24.43 (21.54, 27.33) | 23.15 (18.49, 27.81) |
| Difference | 1.28 (-2.07, 4.62) | -0.72 (-2.86, 1.42) |
| *p-*value | 0.4194 | 0.4658 |
| **Monocyte** |  |  |
| Baseline (Screening) | 7.55 (6.39, 8.71) | 7.57 (6.48, 8.66) |
| week 8 | 8.48 (7.21, 9.75) | 8 (6.68, 9.32) |
| Difference | 0.93 (-0.36, 2.21) | 0.08 (-1.46, 1.62) |
| *p-*value | 0.1404 | 0.9093 |
| **Basophil** |  |  |
| Baseline (Screening) | 0.38 (0.25, 0.52) | 0.41 (0.27, 0.55) |
| week 8 | 0.53 (0.39, 0.67) | 0.34 (0.26, 0.42) |
| Difference | 0.15 (0.01, 0.29) | -0.06 (-0.26, 0.14) |
| *p-*value | 0.2188 | 0.6484 |
| **RBC** |  |  |
| Baseline (Screening) | 5.06 (4.83, 5.30) | 4.89 (4.57, 5.21) |
| week 8 | 5.13 (4.86, 5.41) | 4.86 (4.44, 5.28) |
| Difference | 0.07 (-0.1, 0.24) | -0.07 (-0.27, 0.13) |
| *p-*value | 0.3870 | 0.4499 |
| **Hemoglobin** |  |  |
| Baseline (Screening) | 15.04 (14.18, 15.91) | 13.97 (12.40, 15.53) |
| week 8 | 15.37 (14.44, 16.29) | 13.95 (12.1, 15.8) |
| Difference | 0.33 (-0.04, 0.69) | -0.08 (-0.83, 0.67) |
| *p-*value | 0.0791 | 0.8142 |
| **Hematocrit** |  |  |
| Baseline (Screening) | 45.23 (43.04, 47.41) | 42.77 (38.46, 47.08) |
| week 8 | 46.12 (43.54, 48.69) | 42.65 (37.5, 47.8) |
| Difference | 0.89 (-0.63, 2.41) | -0.49 (-2.37, 1.39) |
| *p-*value | 0.2238 | 0.5704 |
| **Platelet** |  |  |
| Baseline (Screening) | 25.79 (22.64, 28.94) | 27.92 (24.65, 31.19) |
| week 8 | 26.98 (22.7, 31.25) | 28.86 (25.8, 31.92) |
| Difference | 1.18 (-0.91, 3.27) | 0.19 (-1.35, 1.73) |
| *p-*value | 0.2384 | 0.7866 |
| **ESR** |  |  |
| Baseline (Screening) | 4.33 (1.42, 7.24) | 9.17 (3.96, 14.37) |
| week 8 | 5.92 (1.9, 9.93) | 9.6 (4.48, 14.72) |
| Difference | 1.58 (-0.86, 4.03) | 0.1 (-3.04, 3.24) |
| *p-*value | 0.1819 | 0.9441 |
| **AST** |  |  |
| Baseline (Screening) | 18.17 (14.87, 21.46) | 19.58 (15.33, 23.84) |
| week 8 | 26.5 (10.64, 42.36) | 21.6 (15.31, 27.89) |
| Difference | 8.33 (-7.27, 23.93) | 2.3 (-2.37, 6.97) |
| *p-*value | 0.2645 | 0.2944 |
| **ALT** |  |  |
| Baseline (Screening) | 18.17 (11.30, 25.04) | 17.92 (9.03, 26.81) |
| week 8 | 21.92 (13.26, 30.57) | 21.9 (8.89, 34.91) |
| Difference | 3.75 (-0.62, 8.12) | 2.4 (-9.16, 13.96) |
| *p-*value | 0.0855 | 0.6496 |
| **ALP** |  |  |
| Baseline (Screening) | 72.0 (62.79, 81.21) | 74.83 (64.99, 84.67) |
| week 8 | 72.5 (62.63, 82.37) | 79 (70.68, 87.32) |
| Difference | 0.5 (-3.85, 4.85) | 1.8 (-4.34, 7.94) |
| *p-*value | 0.8048 | 0.5240 |
| **Bilirubin Total** |  |  |
| Baseline (Screening) | 0.66 (0.44, 0.87) | 0.71 (0.53 0.89) |
| week 8 | 0.72 (0.57, 0.87) | 0.79 (0.58, 0.99) |
| Difference | 0.06 (-0.11, 0.23) | 0.06 (-0.26, 0.37) |
| *p-*value | 0.4657 | 0.6976 |
| **Na** |  |  |
| Baseline (Screening) | 139.9 (139.4, 140.4) | 140.6 (139.4, 141.7) |
| week 8 | 139.75 (138.93, 140.57) | 139.9 (138.71, 141.09) |
| Difference | -0.17 (-1.06, 0.73) | -0.9 (-2.46, 0.66) |
| *p-*value | 0.6887 | 0.2247 |
| **K** |  |  |
| Baseline (Screening) | 4.36 (4.15, 4.56) | 4.41 (4.27, 4.55) |
| week 8 | 4.33 (4.12, 4.53) | 4.48 (4.2, 4.76) |
| Difference | -0.03 (-0.31, 0.24) | 0.05 (-0.18, 0.28) |
| *p-*value | 0.7949 | 0.6337 |
| **Albumin** |  |  |
| Baseline (Screening) | 4.96 (4.78, 5.14) | 4.82 (4.58, 5.05) |
| week 8 | 4.89 (4.72, 5.06) | 4.88 (4.67, 5.09) |
| Difference | -0.07 (-0.24, 0.11) | -0.01 (-0.24, 0.22) |
| *p-*value | 0.4175 | 0.9246 |
| **Gamma-GTP** |  |  |
| Baseline (Screening) | 25.0 (12.43, 37.57) | 24.08 (10.82, 37.34) |
| week 8 | 25.83 (14.95, 36.72) | 23.4 (10.63, 36.17) |
| Difference | 0.83 (-6.56, 8.23) | -2.8 (-9, 3.4) |
| *p-*value | 0.8087 | 0.3335 |
| **Glucose** |  |  |
| Baseline (Screening) | 96.25 (84.62, 107.90) | 91.42 (85.06, 97.77) |
| week 8 | 99.33 (82.44, 116.23) | 94.6 (87.26, 101.94) |
| Difference | 3.08 (-3.64, 9.8) | 1.3 (-5.05, 7.65) |
| *p-*value | 0.3343 | 0.6545 |
| **BUN** |  |  |
| Baseline (Screening) | 12.53 (10.33, 14.74) | 10.48 (8.96, 11.99) |
| week 8 | 12.46 (10.79, 14.13) | 13.01 (9.66, 16.36) |
| Difference | -0.08 (-2.35, 2.2) | 2.69 (-0.15, 5.53) |
| *p-*value | 0.9434 | 0.0609 |
| **Creatinine** |  |  |
| Baseline (Screening) | 0.82 (0.69, 0.94) | 0.75 (0.64, 0.87) |
| week 8 | 0.8 (0.71, 0.88) | 0.78 (0.65, 0.91) |
| Difference | -0.02 (-0.08, 0.05) | 0.01 (-0.05, 0.06) |
| *p-*value | 0.5669 | 0.8405 |
| **HbA1c** |  |  |
| Baseline (Screening) | 5.53 (5.12, 5.95) | 5.41 (5.25, 5.57) |
| week 8 | 5.61 (5.12, 6.1) | 5.45 (5.28, 5.62) |
| Difference | 0.08 (-0.09, 0.24) | 0.06 (-0.02, 0.14) |
| *p-*value | 0.3509 | 0.1405 |

Values are means (95% confidence intervals).

Supplementary Table S5. Adverse events during the trial period

| Adverse event | Bojungikgi-tang group | Waiting list group | *p-*value* |
| --- | --- | --- | --- |
| Neck and low back pain | 1 | 0 | 0.7602 |
| COVID-19 | 1 | 0 |  |
| Common cold | 3 | 4 |  |
| Cardiovascular sensory abnormalities | 1 | 0 |  |
| Constipation | 1 | 0 |  |
| Chills and fatigue | 1 | 0 |  |
| Elevated fasting blood glucose levels | 1 | 0 |  |
| Elevated AST levels | 1 | 0 |  |
| Elevated WBC and neutrophil levels | 0 | 1 |  |
| **Total** | **10** | **5** |  |

*Fisher's exact test

**Supplementary Figure S1.** **Changes in serum cytokine levels by a multiplex immune-bead assay**

**
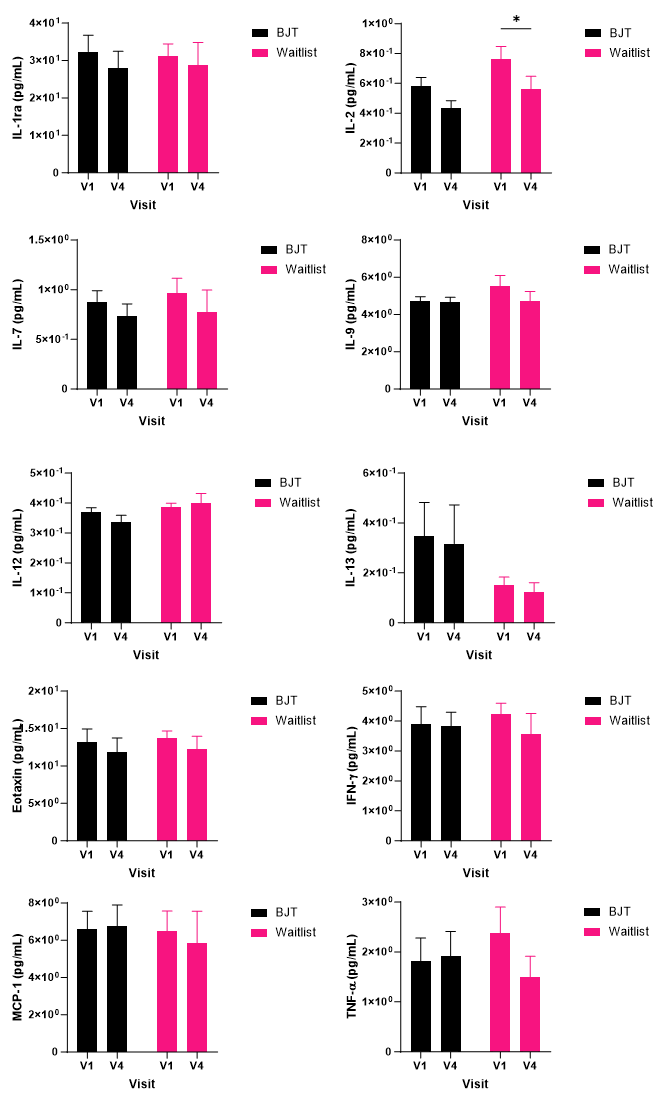
**

BJT, Bojungikgi-tang; IL, interleukin; IFN-γ, interferon-gamma; MCP-1, monocyte chemoattractant protein-1; TNF-α, tumor necrosis factor-alpha; V1, Visit 1 (screening); V4, Visit 4 (week 8).

Data are expressed as the mean ± SEM.

Values below the detection limit were excluded from the analysis. *, p<0.05; paired t-test.

**Supplementary Figure S2. Changes in alpha diversity based on the abundance-based coverage estimator index in the excess and deficiency groups**


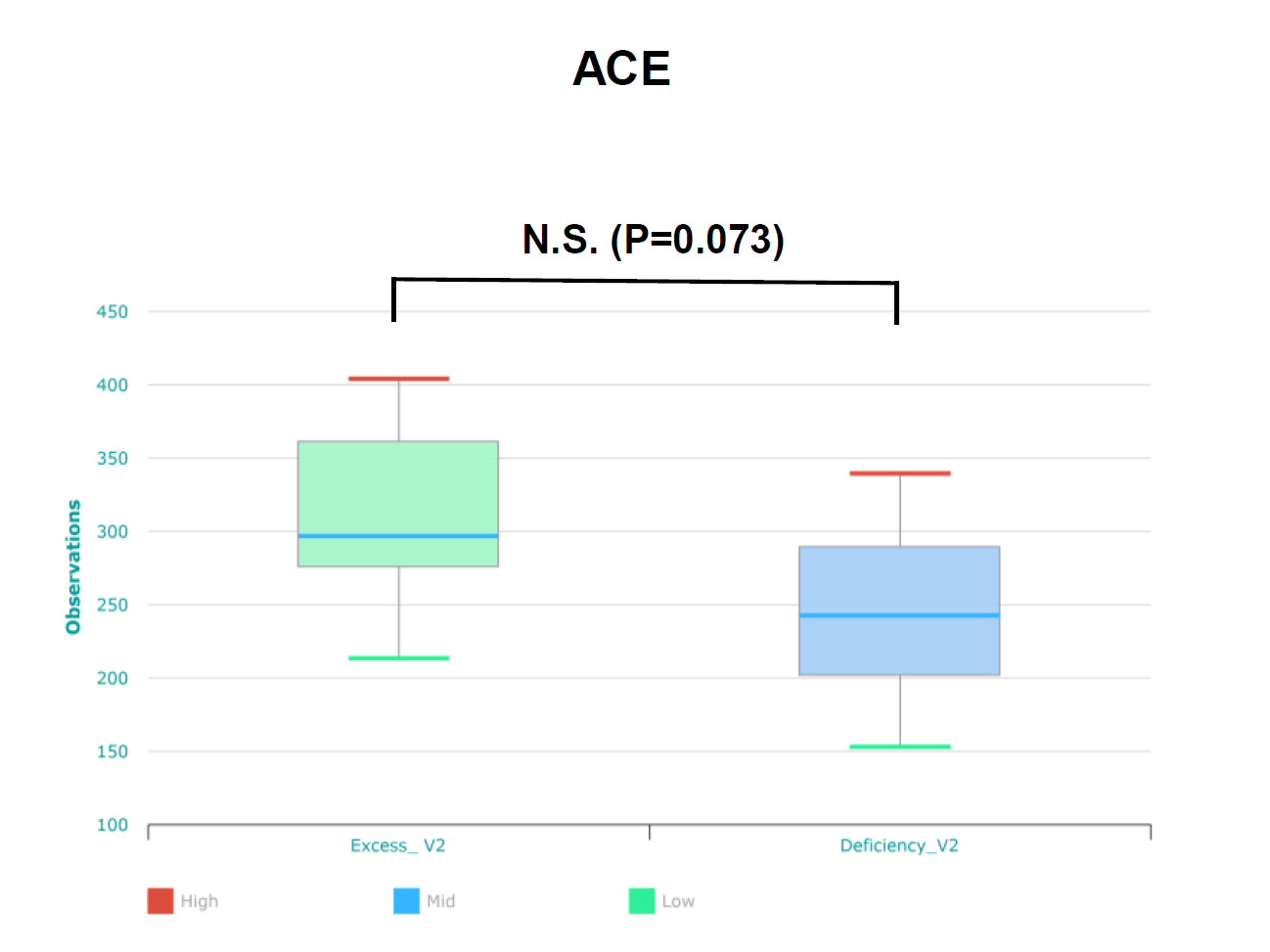


ACE, abundance-based Coverage Estimator; V2, Visit 2 (baseline).

Number of experiments: Excess_V2, n=7; Dificiency_V2, n=14.

**Supplementary Figure S3. The relative abundance of significantly altered fecal microbiota at the species level**


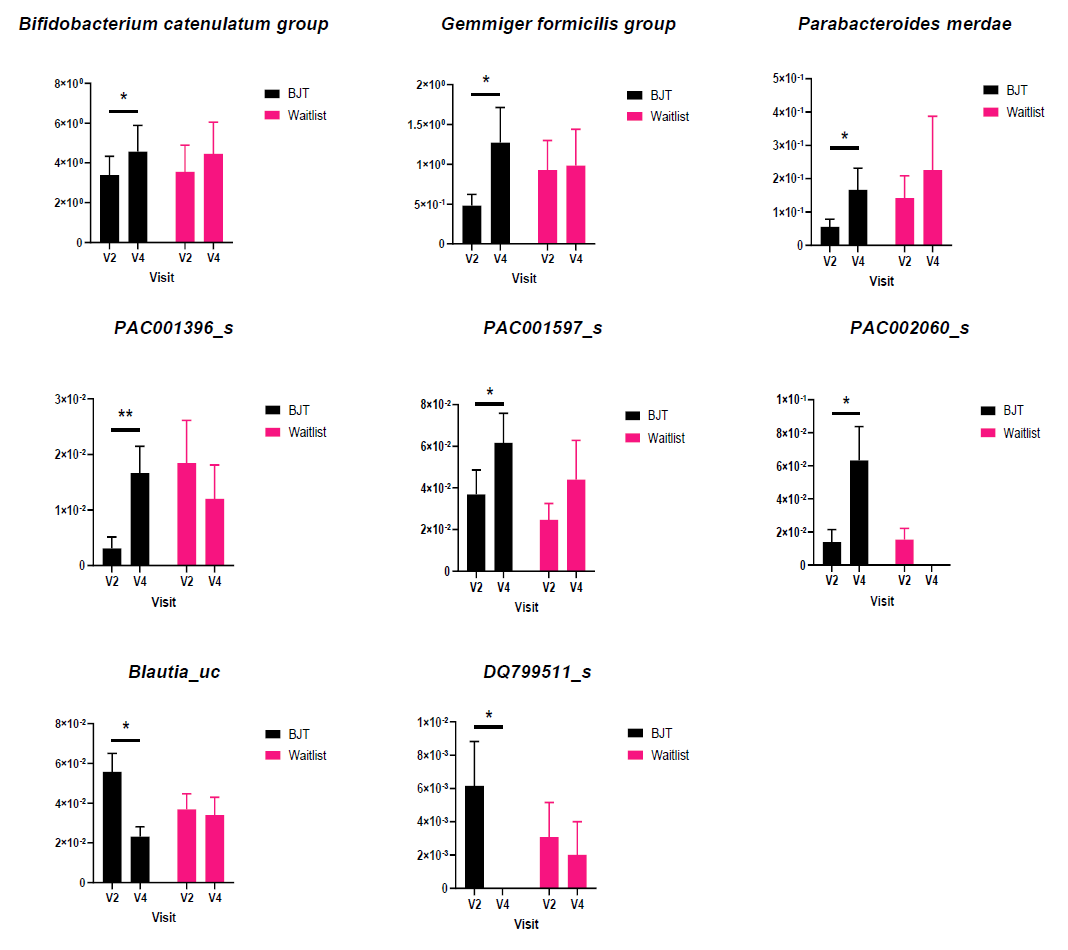


BJT, Bojungikgi-tang; V2, Visit 2 (baseline); V4, Visit 4 (week 8).

Data are expressed as the mean ± SEM.

Number of experiments: BJT_V2, n=13; BJT_V4, n=12; Waitlist_V2, n=13, Waitlist_V4, n=10.

*, p<0.05; **, p<0.01; paired t-test.
